# Diversity, Dispersal and Mode of Reproduction of *Amanita exitialis* in Southern China

**DOI:** 10.3390/genes12121907

**Published:** 2021-11-27

**Authors:** Juan Zhong, Jianping Xu, Ping Zhang

**Affiliations:** 1 College of Life Science, Hunan Normal University, Changsha 410081, China; zhongjuan@hunnu.edu.cn; 2 Department of Biology, McMaster University, Hamilton, ON L8S 4K1, Canada; jpxu@mcmaster.ca

**Keywords:** lethal mushroom, SNP markers, population genetics, secondary homothallism, AMOVA, genetic individual, heterozygosity, Mantel test, genetic clustering, Hardy–Weinberg equilibrium

## Abstract

*Amanita exitialis* is a poisonous mushroom and has caused many deaths in southern China. In this study, we collected 118 fruiting bodies of *A. exitialis* from seven different sites in Guangdong Province in southern China and investigated their genetic relationships using 14 polymorphic molecular markers. These 14 markers grouped the 118 fruiting bodies into 20 multilocus genotypes. Among these 20 genotypes, eight were each found only once while the remaining 12 were each represented by two to 54 fruiting bodies. Interestingly, among the 12 shared genotypes, four were shared between/among local populations that were separated by as far as over 80 km, a result consistent with secondary homothallic reproduction and long-distance spore dispersal. Despite the observed gene flow, significant genetic differentiations were found among the local populations, primarily due to the over-representation of certain genotypes within individual local populations. STRUCTURE analyses revealed that the 118 fruiting bodies belonged to three genetic clusters, consistent with divergence within this species in this geographic region. Interestingly, we found an excess of heterozygous individuals at both the local and the total sample level, suggesting potential inbreeding depression and heterozygous advantage in these populations of *A. exitialis*. We discuss the implications of our results for understanding the life cycle, dispersal, and evolution of this poisonous mushroom.

## 1. Introduction

Over the last decade, more than 10,000 mushroom poisonings have been reported in China, including at least 788 deaths. In addition, from 2010 to 2020, the number of recorded mushroom poisoning has increased significantly, from 37 in 2010 to 2,705 in 2020 [1]. In China, 480 mushroom taxa are known to be poisonous [2], among which 12 belonged to the genus *Amanita* [3,4,5]. These 12 *Amanita* species contain the lethal amanitin toxins and are among the deadliest mushrooms known. From 1994 to 2012, more than 90% of fatal mushroom poisonings were caused by ingestion of lethal amanitas in Australia, East Asia, and North America [6,7,8,9]. Investigations from 2000 to 2019 in southern China indicated that *Amanita exitialis* Zhu L. Yang & T.H. Li caused at least 116 poisoning cases including 44 fatalities, accounted for 73.33 % of the mortality from lethal mushroom poisoning cases in the area [6,10,11,12]. The early reports of *A. exitialis* poisoning mainly came from Guangdong Province in southern China [13]. However, in the last few years, documented *A. exitialis* poisoning cases have been reported in Yunnan and Guizhou Provinces in China as well as in Thailand [14,15,16]. The toxins in *A. exitialis* include α-amanitin (α-AMA), β-amanitin (β-AMA), phallacidin (PCD), phallisacin (PSC), phallisin (PHS) and phalloin (PHN), with the highest content being α-AMA [17,18,19]. Ingestion of these toxins can lead to acute liver failure and death [13,17,18].

The fungus *A. exitialis* was first described in China by Yang and Li in 2001 [20] and was subsequently reported from India and Thailand [21]. This species belongs to the lethal *Amanita* subclade in *Amanita* section *Phalloideae* (Fr.) Que´l. Morphologically, the fruiting bodies are white for both the cap and stipe, with the center of the cap often appearing creamy. The fruiting bodies are typically small to medium-sized with pileus 4 to 8 cm in diameter, with a friable annulus and a subglobose basal bulb. The basidiospores of *A. exitialis* are relatively large, 9.5–12.0 × 9.0–11.5 µm, globose to subglobose in shape, and amyloid. Unlike most agaric mushrooms which typically have four-spored basidia, the basidia of *A. exitialis* are almost entirely two-spored [3]. It is noteworthy that *A. exitialis* forms basidiomata in early spring to early summer in Guangdong Province but in middle to late summer in Yunnan Province [4]. Field observations suggested that *A. exitialis* associates mycorrhizally with *Castanopsis fissa* (Champ. ex Benth.) Rehd. et Wils. or *Lithocarpus glaber* (Thunb.) Nakai, two deciduous Fagaceae tree species found only in East Asia and South Asia. 

Like most mushrooms, the fruiting bodies of *A. exitialis* represent only the sexual reproductive structure while most of the biomass of the fungus in nature are present underground as vegetative mycelia. Fruiting bodies at close proximity to each other but sharing the same genotype could be derived from the same vegetative mycelia, from the same ancestral spore/mating event and represent the same genetic individual of *A. exitialis* in nature. Thus, the relationship between physical distance and genotypes among the fruiting bodies within each site could help shed light on the potential size and longevity of the genetic individuals of this species in nature. At present, the size of genetic individuals of *A. exitialis* in nature is not known.

Several recent studies of *A. exitialis* have greatly contributed to our understanding of this species at the molecular level. For example, the transcriptome data for *A. exitialis* were obtained in 2013 [22,23]. Comparative genome analyses with other poisonous amanitas helped researchers identify the genes responsible for α-AMA biosynthesis in *A. exitialis* fruiting bodies [24], as well as MSDIN genes and the gene coding for prolyl oligopeptidase B (POPB) [22]. Based on the transcriptome sequence, He et al. designed MSDIN and POPB gene-specific primers for rapid assays of toxic mushrooms in 2019 [25]. However, there has been no research on the patterns of genetic variation in natural populations of *A. exitialis*. 

In this study, we collected and analyzed fruiting bodies of *A. exitialis* from seven sites in Guangdong Province in southern China. These samples were used to address several issues. First, are local and regional populations genetically differentiated, or are gene flows abundant among local and regional populations? Second, do all strains in the currently recognized *A. exitialis* species belong to one inter-breeding population in nature, or are there differentiated genetic clusters within this species from Guangdong Province? Third, what’s the likely mode of reproduction for *A. exitialis* in nature? Is it a homothallic, secondary homothallic, or heterothallic species?

## 2. Materials and Methods

### 2.1. Materials

**Figure 1:**
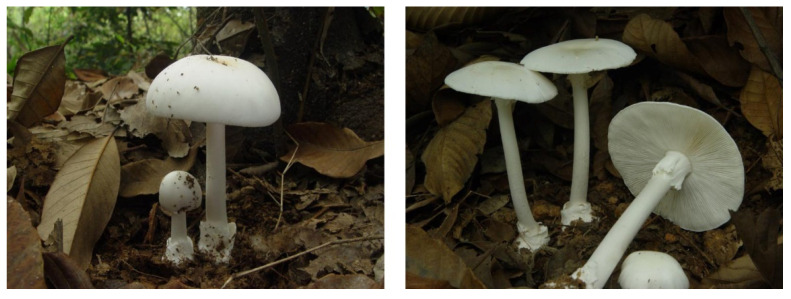
The basidiocarps of *A. exitialis*.

The fruiting bodies of *A. exitialis* were collected over a five year span, from 2007 to 2012 in Guangdong Province. In total, we collected 118 fruiting body samples of *A. exitialis* from seven sites. Figure 1 shows the typical fresh basidiocarps of *A. exitialis*. Information about the seven collection sites is shown in Table 1.

### 2.2. DNA Extraction, Primer Selection and SNP Markers

To screen for polymorphic molecular markers, we first extracted the genomic DNA of a representative fruiting body of *A. exitialis*, using the CTAB-based method that was slightly modified for fungi as described previously [26]. The genomic DNA was digested by the *Hae*III endonuclease and run on 1% agarose gel [27]. The DNA fragments between 1kb to 1.5kb were excised from the agarose gel and purified with the QIAquick PCR purification Kit. The obtained DNA fragments were subcloned into pPCR-Script Amp predigested cloning vector with a PCR-Script Amp Cloning Kit (Stratagene, La Jolla, CA, USA). The recombinant molecules were transformed into the XL10-Gold® Kan Ultracompetent cells. The transformed cells were grown and screened for *E. coli* colonies containing recombinant molecules. The *A. exitialis* genomic DNA fragments inserted into the cloning vector were individually amplified by PCR and sequenced.

From the obtained *A. exitialis* DNA sequences, we designed a total of 80 primer pairs to screen for DNA fragments with readily recognizable sequence polymorphisms, including single nucleotide polymorphisms (SNPs) and insertion/ deletions, within the same fruiting body that we used to construct the genomic library. Among these 80 primer pairs, six pairs amplified fragments containing sequence polymorphisms within the mushroom. The SNPs within the fruiting body were shown as double peaks in the sequence chromatograph at specific positions. In contrast, the insertions/deletions were shown as either amplified fragment length polymorphisms on the agarose gel (large insertion/deletions) or in chromatographs where the 5’ portion of the sequence was clean while the remaining portion towards the 3’ end after the insertion/deletion was unreadable. The sequences for the six primer pairs are as follows (5’-3’): (1) A31-F GGGCGCAGTGGAATAGTAGA, A31-R TCCACAGATGGATGGACAAA; (2) G24-F CCTCTGGAGAAGTGGTTGGA, G24-R GGATGCAGCCTTACTGGAAA; (3) C21-F: TATCCAAAGACGTCCCCTCA, C21-R CGATTTTCCAGCTCGCTAAC; (4) H11F: GAGATGCGAGAGATCATACCG, H11R: GGTTCAGGTTCGTCCGAGT; (5) K47-F CGGAGTGGAACATGGTGTATC, K47-R: GCACTCCCACACCTCAACTT; (6) M12-F: GGGTGGGCAACTCTCTGTTA, M12-R AGGAGCTAGGGCTCAACTG. These six primer pairs were used to amplify and sequence all 118 fruiting bodies of *A. exitialis*. A total of 482 sequences and amplified fragment length polymorphisms shown on agarose gels (after electrophoresis) from these samples were analyzed to identify the patterns of genetic variation within and among fruiting bodies and populations of *A. exitialis* in Guangdong Province.

### 2.3. Data and Statistical Analysis 

#### 2.3.1. Analyses of Molecular Variance (AMOVA)

The program GenAlEx v6.5 was used to infer the relationships among local populations through the analysis of molecular variance (AMOVA) based on the observed DNA polymorphisms data [28]. In addition, the potential relationship between genetic distance and geographic distances among fruiting bodies from the seven geographical populations was examined using a Mantel test [29] as implemented in GenAlEx.

#### 2.3.2. STRUCTURE for Population Genetic Structure Analysis

To identify whether our samples contain divergent genetic clusters of *A. exitialis*, we analyzed our *A. exitialis* samples using the program STRUCTURE v2.3.4, based on an admixture model. Models were tested for *K*-values ranging from 2 to 7, with 10 independent runs per *K* value. For each run, the initial burn-in period was set to 100,000 with 1,200,000 MCMC iterations. To determine the most probable value of *K*, the delta *K* method was used and implemented in Structure Harvester [30,31]. In addition, Distruct1.1 [32] and CLUMPP v1.1.2b [33] were used to infer the optimal *K*-cluster affiliations of individual fruiting bodies and populations based on STRUCTURE results respectively. The geographic map of *A. exitialis* fruiting bodies and populations was generated with ArcGIS v10.0 (http://www.esri.com/software/arcgis/arcgis-for-desktop, accessed on 1 November 2021) and modified manually by highlighting different landscape features and different genotypes with different colors using Adobe Photoshop CS6 (http://www.adobe.com/products/photoshop, accessed on 1 November 2021). 

#### 2.3.3. Genetic Relationships among Fruiting Bodies

To illustrate the relationships among the fruiting bodies based on their genotypes at the 14 molecular markers, we used the program MEGA v7 based on the Neighbor-Joining algorithm [34,35]. The potential migration among the seven geographical populations was detected by the Treemix program based on allele counts at these marker loci [36]. The specimen collection diagram uses Adobe Photoshop CS6 and Google Maps, and the colors are marked based on the population genetic structure data. Furthermore, the software OriginPro2018 (https://www.originlab.com/, accessed on 28 October 2021) was used to identify genetic clusters among individuals, which were then transformed into a three-dimensional principal components analysis (PCA) plot [37].

#### 2.3.4. Mode of Reproduction in Nature

To investigate the potential mode of reproduction of *A. exitialis*, we compared the observed and expected counts of genotypes at each of the 14 molecular markers. The expected counts were derived based on the observed allele frequencies at each of the 14 markers and assuming random mating. Specifically, if there is random mating in the population, we expect that the observed genotype frequencies will be in Hardy–Weinberg equilibrium. Rejection of the Hardy–Weinberg equilibrium hypothesis would be inconsistent with random mating. Tests of Hardy–Weinberg equilibrium were conducted using the GenAlEx 6.5 program.

In addition to the test of Hardy–Weinberg equilibrium, we also examined the relationship between physical distance and genotypes of our fruiting bodies within each of the seven sites to determine the potential size of genetic individuals at each site. 

## 3. Results

In this study, we developed 14 molecular markers and used these markers to analyze their patterns of genetic variation within and among 118 fruiting bodies of the poisonous mushroom *A. exitialis* from seven sites in Guangdong Province in southern China. Our genetic analysis grouped the 118 fruiting bodies into 20 multilocus genotypes. Below we describe the observed population genetic variation within and among the seven sites.

### 3.1. Distributions of Multilocus Genotypes within and among Sites

**Figure 2:**
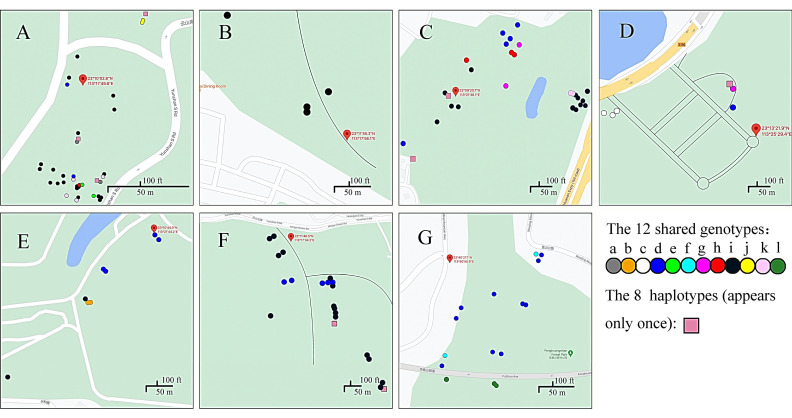
Distributions of multilocus genotypes within and among the seven collection sites. Sub-figures A to G illustrate the spatial distribution of individual fruiting bodies and their multilocus genotypes in site A to G. Filled circles of the color represent the same multilocus genotype: site A contains 36 fruiting bodies belonging to nine genotypes; site B contains four fruiting bodies belonging to one genotype; site C contains 27 fruiting bodies belonging to seven genotypes; site D contains six fruiting bodies belonging to four genotypes; site E contains eight fruiting bodies belonging to three genotypes; site F contains 22 fruiting bodies belonging to four genotypes; and site G contains 15 fruiting bodies belonging to three genotypes. The geographic location of sites A to G are shown in Table 1.

The distribution of the 20 multilocus genotypes is shown in Figure 2. Among these 20 genotypes, eight were unique, with multilocus genotype represented by only one fruiting body each. These eight multilocus genotypes are represented by pink squares in Figure 2. Among these eight genotypes, three were found in site A, two were in site C, one was in site D, and two were in site F. The remaining 12 genotypes were shared by two to 54 fruiting bodies distributed within and among the seven sites. 

Here, the 12 shared genotypes are labeled as “a, b, c, d, e, f, g, h, i, j, k, l”, and they are represented by circles with different colors (Figure 2). Among these 12 shared genotypes, seven (“a”, “b”, “c”, “e”, “f”, “j”, “l”) only appeared in one local population each (the numbers of fruiting bodies for these seven shared genotypes are as follows: “a” = 2, “b” = 2, “c” = 3, “e” = 2, “f” = 2, “j” = 2, “l” = 3). Of the remaining five, genotype “d” contained 27 fruiting bodies distributed in six sites A, C, D, E, F, and G; genotype “g” contained three fruiting bodies distributed in two sites C and D; genotype “h” contained four fruiting bodies distributed in two sites A and C; genotype “i” contained 54 fruiting bodies distributed in five sites A, B, C, E, and F; and genotype “k” contained six fruiting bodies distributed in two sites A and C.

Overall, among the seven local populations, a range of one to nine genotypes was found at each site. Specifically, nine genotypes were found in site A, one in site B, seven in site C, four in site D, three in site E, four in site F, and three in site G. 

Based on the distributions of shared genotype(s) within each of the sites, we estimated the approximate size of each genetic individual. As described above, among the 12 shared genotypes, seven (“a”, “b”, “c”, “e”, “f”, “j”, “l”) were found shared among fruiting bodies from within the same site. Their maximum physical distance between the two furthest fruiting bodies ranged from 10m (genotype “b” at site E and genotype “j” at site A) to 200 meters (genotype “f” at site G) (Figure 2). However, among sites, the physical distance between the fruiting bodies with shared genotypes ranged from 8km (genotype “g”) to over 80 kilometers (genotype “d”).

### 3.2. AMOVA Analysis and Mantel Test Results

For the seven natural geographical populations of *A. exitialis*, the analysis of molecular variance (AMOVA) showed that most (81%) of the observed genetic variations were found within individual local populations. However, 19% of total genetic diversity was attributable to among-population variation (*p* < 0.01; Table 2), consistent with significant genetic differentiation among these seven local populations. Despite the observed statistically significant differentiation, the estimated overall gene flow among the seven local populations was greater than 1 (*N_m_* = 1.056), suggesting that the local populations are unlikely to be completely differentiated into different geographic species.

**Figure 3:**
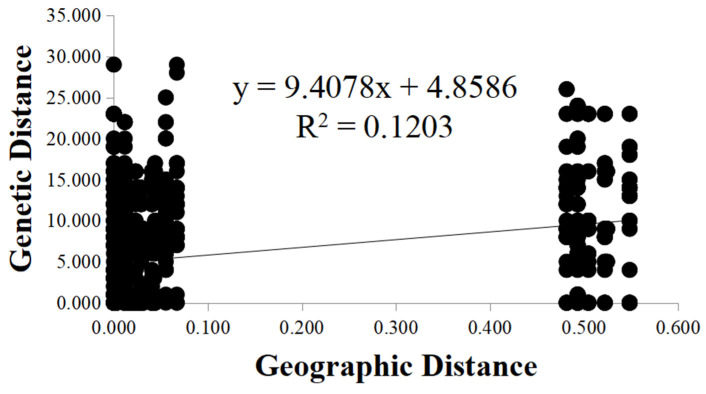
Mantel test between genetic distance and geographic distance among the *A. exitialis* populations.

To investigate whether the geographic distance was a contributor to the observed genetic differentiation among the seven local populations, we determined the relationship between geographic distance and genetic distance between all pairs of fruiting bodies analyzed here using the Mantel test. Our analysis indicated a positive correlation between the genetic distance and geographic distance among the 118 fruiting bodies at the seven sites (*R*^2^ = 0.1203, *p* = 0.001) (Figure 3). The results suggested that physical distance was an important factor shaping the genetic structure among local populations of *A. exitialis*. 

### 3.3. Genetic Clustering

**Figure 4:**
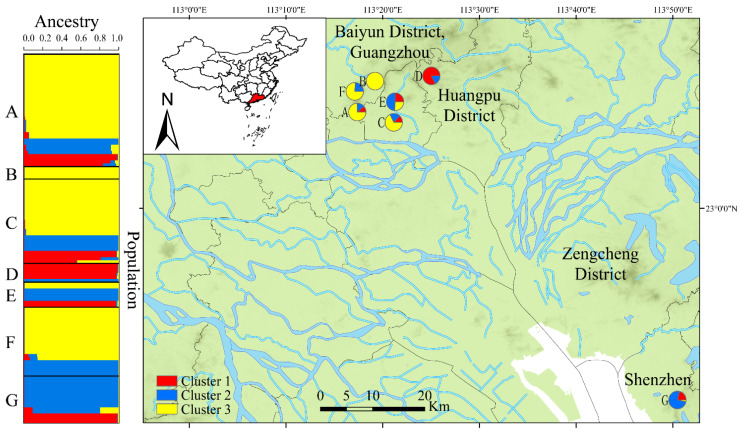
Locations and genetic structure of seven populations of *A. exitialis*. STRUCTURE clustering analysis results based on their geographic distribution on the left. Codes of the populations are given in Table 1.

The STRUCTURE program was used to infer the potential number of genetic clusters in our sample. Our analysis identified a strong peak of Δ*K* = 3, which indicated that the 118 fruiting bodies of *A. exitialis* were optimally clustered into three groups which we defined them as “Cluster 1” (in red), “Cluster 2” (in blue), and “Cluster 3” (in yellow) (Figure 4). Overall, Cluster 3 contained the highest number of fruiting bodies, followed by Cluster 2 and Cluster 1. Except for site B which contained only four fruiting bodies with all four belonging to Cluster 3, the remaining six local geographic populations each contained genetic elements from at least two clusters. Among the seven local populations, four local populations were dominated by genotypes in Cluster 3 (with all four in Guangzhou); one each in Guangzhou and Shenzhen were dominated by Cluster 2 genotypes, and the last local population in Guangzhou was dominated by Cluster 1 genotypes (Figure 4).

### 3.4. Neighbor-Joining (NJ) Phylogenetic Tree and PCA Analysis

**Figure 5:**
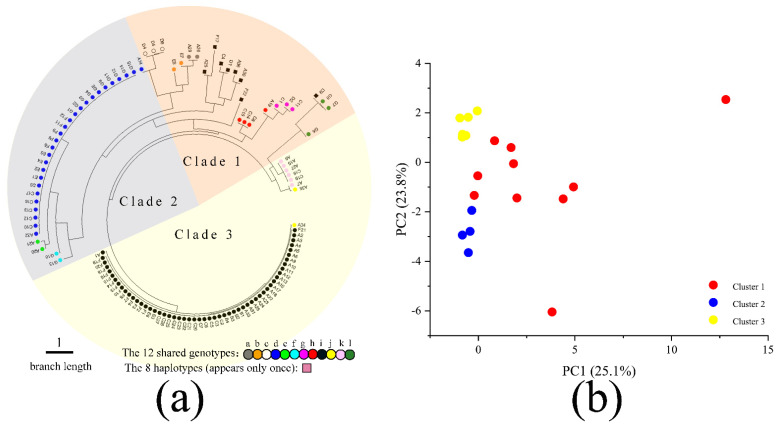
(**a**). Dendrogram obtained for the 118 *A. exitialis* fruiting bodies based on their genetic distance; (**b**). The hierarchical clustering results of 118 *A. exitialis* fruiting bodies based on principal component analyses.

To analyze the genetic relationships among the 118 *A. exitialis* fruiting bodies, we used the linear genetic distance based on their allelic composition at the 14 marker loci to build a neighbor-joining dendrogram (Figure 5a). The dendrogram results are largely consistent with those inferred based on the STRUCTURE program. Here, three fan-shaped colors correspond to the three genetic clusters as inferred based on the STRUCTURE program. Clade 1 is marked by the pale red fan-shape and it contains 25 fruiting bodies. Clade 2 is marked by the pale-blue fan-shape and it contains 31 fruiting bodies. Clade 3 is marked by the pale-yellow fan-shape and it contains 62 fruiting bodies. At the genotype level, the shared genotypes “d”, “e” and “f” are found in Clade 2. Shared genotypes “i”, “j” and “k” are found in Clade 3. The remaining shared genotypes as well as the eight unique genotypes (i.e., those represented by only one fruiting body each) are mainly found in Clade 1. As indicated above, the shared genotypes “d” and “i” were the most frequently found genotypes, represented by 27 and 54 fruiting bodies respectively. 

Similarly, principal components analysis (PCA) showed the separation of the 118 fruiting bodies into three groups on this two-dimensional space (Figure 5b). Individuals in Cluster 2 and Cluster 3 are grouped relatively tightly within each group, while some individuals in Cluster 1 are relatively scattered. Overall, results from the neighbor-joining dendrogram, PCA, and STRUCTURE analyses are largely consistent with each other. However, inconsistencies were also observed. For example, phylogenetically, strains D4, D5, and D6 were close to A14 but they belonged to two different genetic clusters (Figure 5a).

### 3.5. Hardy–Weinberg Equilibrium Test

Based on the genotypes and allele frequencies in our sample of the 118 fruiting bodies of *A. exitialis*, we assessed whether the total population was in Hardy–Weinberg equilibrium (HWE). If the total population was randomly mating, we expected that the population would be in HWE, with the observed heterozygosity not significantly different from the expected heterozygosity at each locus. Our results rejected the null hypothesis of HWE for the total population at each of the 14 marker loci (Table 3). Interestingly, among the 14 loci, nine showed excess observed heterozygosity over expected heterozygosity while five loci showed the opposite pattern (Table 3).

## 4. Discussion

Each year, poisonous mushrooms kill hundreds of people worldwide. However, little is known about the population biology and mode of reproduction of these mushrooms. In this study, we analyzed the fine-scale genetic variation of the poisonous mushroom *A. exitialis* from Guangdong Province in southern China. Our results revealed a relatively limited number of genotypes, much lower than would be expected under the assumption of random mating and independent assortment among the six amplified DNA fragments. Interestingly, we found significant genetic differentiation among the local geographic populations. Furthermore, the analyzed samples were grouped into three genotype clusters which were broadly supported by different analytical methods. However, there was also abundant genotype-sharing among the local sites. Below we discuss the implications of our results for understanding the evolution and mode of reproduction of *A. exitialis* in nature.

The fixation index *F_st_* and *N_m_* (number of migrants) are important indicators for genetic, structural and gene flow analyses among geographic populations. Based on Wright’s finite island model of population structure, *N_m_* = [(1/*F_st_*) – 1]/4, *F_st_* values of 0.05–0.15, 0.15 < *F_st_* < 0.25, and >0.25 are indicative of moderate, high, and very high genetic differentiation among populations, respectively. If neutral genetic markers are used and assuming constant population size, the degree of genetic differentiation among geographic populations would be closely related to gene flow and to genetic drift within and between populations. In general, *N_m_* values > 1.0 indicate that the populations are unlikely to be completely differentiated. When *N_m_* < l.0, increasing genetic differentiation among populations over time is expected due to genetic drift [38,39], and local selection would accelerate the differentiation among sites. In the case of *A. exitialis*, the observed *F_st_* value was 0.19, corresponding to a high level of genetic differentiation among the local populations. On the other hand, shared genotypes were common among the sites. However, the shared genotypes were not uniformly distributed among different local populations. Instead, differential over-representation of certain genotypes was common in each of the seven local populations, causing significantly biased allele frequencies for one or the other allele at each of the 14 marker loci and contributing to the high-level differentiation among the local populations. Indeed, if clone-corrected data were used, the overall *F_st_* value would decrease drastically to <0.05 (due to the small sample sizes after clone correction at all sites, details of the clone-corrected analyses results are not shown). At present, the reasons why certain multilocus genotypes are more broadly distributed than other genotypes are not known. However, both the age of the founding population and the location-specific selection forces could have contributed. For example, an older founding population and a more favorable mushroom growth and fruiting environment may lead to the production of more fruiting bodies from a bigger genetic individual with the same genotype. 

**Figure 6:**
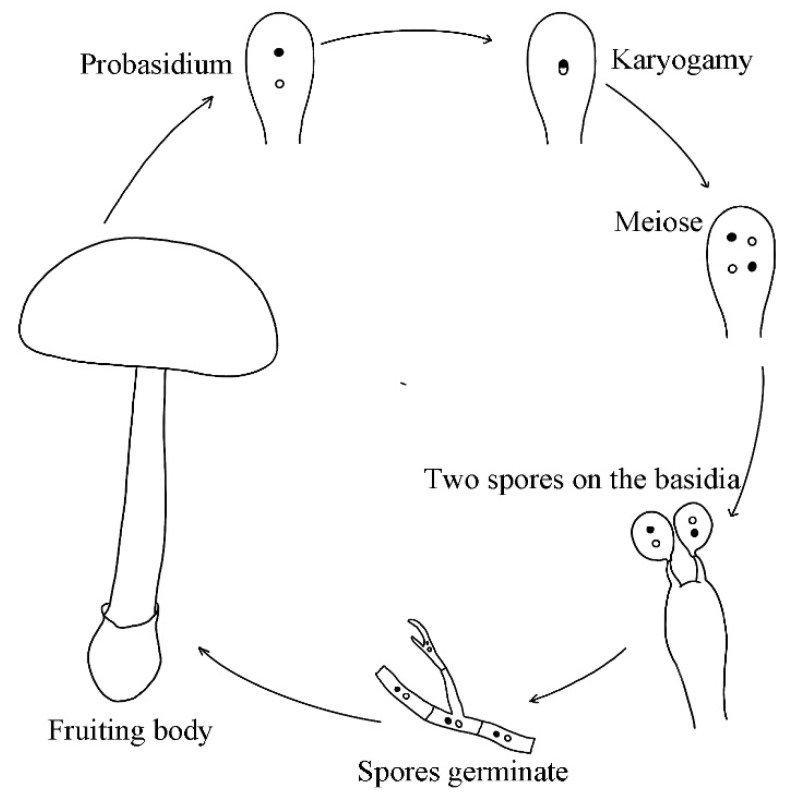
A proposed model for the most likely mode of reproduction for *A. exitialis* in nature.

Based on the observed basidia structure where most basidia had only two basidiospores each, the most likely mode of reproduction is secondary homothallism, similar to that of the commercial button mushroom *Agaricus bisporus* var. *bisporus* [40]. In secondary homothallic reproduction, most basidia produce two basidiospores each and each basidiospore receives two nuclei that are non-sisters to each other. Consequently, the two nuclei within each basidiospore likely have different mating types and such basidiospores would be capable of completing the sexual reproductive life cycle and forming a fruiting body all by itself without the need for mating. Furthermore, if recombination frequency is low between homologous chromosomes, these basidiospores will retain (almost) all alleles of the parental nuclei, maintaining the parental genotype [41]. These basidiospores could be dispersed by wind or by anthropogenic activities. Based on the observations, we propose the following model for the most likely mode of reproduction for *A. exitialis* in nature (Figure 6).

However, we should note that if secondary homothallism was the only mode of reproduction, heterozygosity would likely decrease over time due to meiotic crossing over and segregation during basidiospore formation, causing lower observed heterozygosity than expected heterozygosity over time. However, as was shown in Table 3, nine of the 14 marker loci showed excess heterozygosity. At present, the mechanism for the observed excess heterozygosity at the nine marker loci is not known. There are three non-exclusive possibilities. In the first, the mutation rate in this species might be higher than the recombination rate, leading to the accumulation of genetic variations within individual genomes in certain genetic individuals. A similar argument was recently presented to explain the human opportunistic fungal pathogen *Candida tropicalis* [42]. In the second, heterozygotes are more advantageous than homozygous in these environments, causing selective elimination of homozygotes from natural environments. Indeed, evidence for excess heterozygosity and inbreeding degression has been reported in the secondary homothallic mushroom *A. bisporus* [43,44]. In the third, there may be rare homokaryotic self-sterile basidiospores in *A. exitialis* that upon germination, can mate with homokaryons with compatible mating types to form fertile heterokaryons and increase heterozygosity. All three mechanisms could contribute to the heterogeneous distribution of heterozygosity in the natural population of *A. exitialis*, with excess heterozygosity in certain regions of the genome while lower than expected heterozygosity in other parts of the genome.

**Figure 7:**
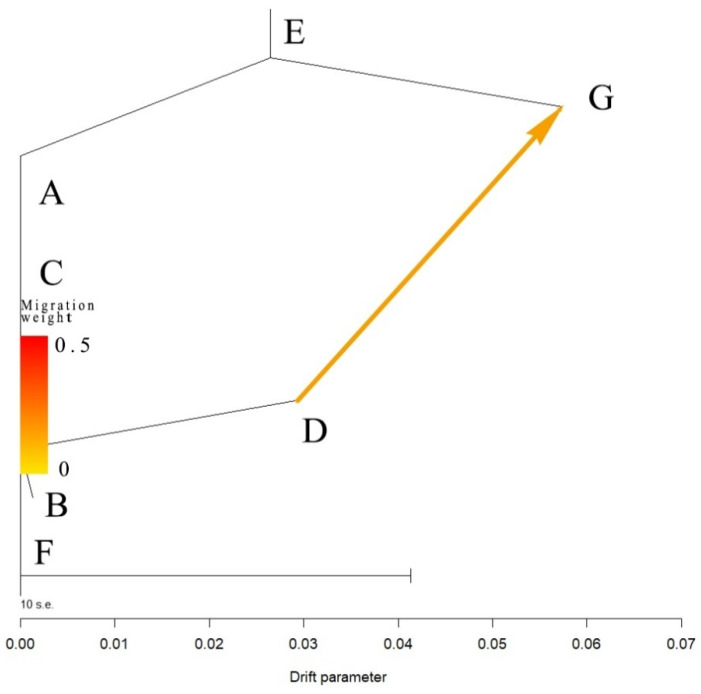
Detection of gene flows among *A. exitialis* populations by TreeMix analysis.

The geographical distribution of genotypes provides important information for understanding the biogeography and evolution of populations and species, including for tracking the origin and dispersal routes of specific genotypes and lineages. Between Guangzhou city and Shenzhen city, there are several mountains, rivers, densely populated cities, and other geographic barriers. However, in our study, one genotype “d” was broadly shared between these two cities separated by over 80 kilometers. Both wind-aided dispersal and anthropogenic activities could have facilitated the dispersal of self-fertile basidiospores between the two cities. In this case, the TreeMix analysis revealed that the dispersal was most likely from site D in Guangzhou to site G in Shenzhen (Figure 7). However, more sampling between these two cities and more detailed genotype analyses, such as through whole-genome sequencing is needed to identify whether this genotype (and potentially other genotypes) is broadly distributed throughout Guangdong Province and if so, what the likely colonization and dispersal history of this genotype might have been in this geographic region. 

In Guangdong Province, *A. exitialis* is commonly associated with *Castanopsis fissa*, an evergreen tree in Fagaceae that is broadly distributed in southern China and Southeast Asia. This evergreen tree species has been considered the host species of *A. exitialis*. However, in most of the seven sites, fruiting bodies of *A. exitialis* were found growing under a mixture of trees, including a different evergreen species, *Lithocarpus glaber*. *L. glaber* is distributed in Japan and China. At present, the importance of other trees, such as *L. glaber* in the growth and reproduction and in ectomycorrhizal associations with *A. exitialis* awaits further investigation.

## 5. Conclusions

In this study, our focus was on spatial distributions of *A. exitialis* genotypes. Temporally, both identical and different genotypes of *A. exitialis* were found within the same site between years during our sampling period from 2007 to 2012. This is not surprising given the secondary homothallic reproduction of this species and its ectomycorrhizal association with trees that may last many years. Our results are different from those in *Amanita manginiana* where no identical genotype was found between two years of sampling at a site in Sichuan Province, southwestern China [45]. The reason for the difference between these two species was likely due to their different reproductive life cycles. *A. manginiana* is a heterothallic species with four spores per basidia, as well as has small and short-lived genetic individuals in nature. In contrast, as shown here, *A. exitialis* has a secondary homothallic life cycle and likely has large long-lived genetic individuals. The spatial and population genetic features of *A. exitialis* identified in our study could help prevent future mushroom poisoning. For example, warning signs containing pictures of *A. exitialis* fruiting bodies should be installed at specific locations within each site to explicitly warn people to not pick this poisonous mushroom.

## Figures and Tables

**Table 1:** *Amanita exitialis* sample collection information.

Code	City	Location	Sample Size	Latitude	Longitude	Altitude (above Sea Level)
A	Guangzhou	Duguting, Baiyunshan Mountain	36	23.1674292	113.2971586	200–300 m
B	Guangzhou	Guangzhou University of Foreign Studies	4	23.1986838	113.2989164	0–50 m
C	Guangzhou	South China Agricultural University	27	23.1557492	113.3600235	0–50 m
D	Guangzhou	Guangzhou Tianluhu Forest Park	6	23.2227467	113.4248414	200–300 m
E	Guangzhou	South China Botanical Garden	8	23.1788847	113.3622869	0–50 m
F	Guangzhou	BeeWorld, Baiyunshan District	22	23.1963429	113.2928271	100–200 m
G	Shenzhen	Fenghuangshan Forest Park	15	22.6742000	113.8488670	100–200 m

**Table 2:** AMOVA of genetic variation among seven populations of *A. exitialis*.

Source of Variation	d.f.	Sum of Squares	Variance Components	Percentage of Variation (%)	*F_st_*	*N_m_*
Among-population	6	71.906	11.984	19%	0.191 ***	1.056
Within-population	111	282.780	2.548	81%		
Total	117	354.686		100%		

*** *p* < 0.01.

**Table 3:** Chi-Square Tests for Hardy–Weinberg Equilibrium using the total *A. exitialis* sample.

Locus	Observed Heterozygotes	Expected Heterozygotes	Chi-Square Value
M12	73	49.640	21.849 ***
C21	106	59.000	74.881 ***
H11-300	64	53.809	5.104 *
A31-88	0	5.847	118.000 ***
A31-138	0	1.983	118.000 ***
A31-425	3	46.182	103.167 ***
A31-440	71	55.436	9.301 **
G24-106	6	11.390	26.424 ***
G24-163	41	33.877	5.217 *
G24-489	77	51.877	27.674 ***
G24-600	106	58.390	78.453 ***
G24-615	5	10.487	32.305 ***
K47-355	103	58.962	65.826 ***
K47-556	103	58.962	65.826 ***

* *p* < 0.05, ** *p* < 0.01, *** *p* < 0.001.

## Data Availability

Not applicable.
